# Impact of hepatic venous oxygen efflux and carotid blood flow on the difference between mixed and central venous oxygen saturation

**DOI:** 10.1186/cc9455

**Published:** 2011-03-11

**Authors:** T Correa, R Kindler, S Brandt, J Gorrasi, T Regueira, H Bracht, F Porta, J Takala, R Pearse, S Mathias Jakob

**Affiliations:** 1 University Hospital Bern - Inselspital and University of Bern, Switzerland; 2 Royal London Hospital, London, UK

## Introduction

The difference between central venous (ScvO_2_) and mixed venous oxygen saturation (SvO_2_) may vary widely. The objective of this study was to evaluate the impact of hepatic and renal venous oxygen efflux, femoral oxygen saturation and carotid artery blood flow on the difference between ScvO_2 _and SvO_2 _(Δ[ScvO_2 _- SvO_2_]).

## Methods

Nineteen sedated and mechanically ventilated pigs (weight: 41.0 ± 3.6 kg) were subjected to sepsis (*n *= 8), hypoxic hypoxia (*n *= 3) and cardiac tamponade (*n *= 3) or served as controls (*n *= 5). Mixed, central and regional venous oxygen saturations (spectrophotometry), and carotid, hepatic and renal blood flows (ultrasound Doppler flow) were measured at baseline and 3 hourly, up to 24 hours. Hepatic venous oxygen efflux was determined as hepatic arterial + portal venous blood flow times hepatic venous oxygen content, and renal venous oxygen efflux as twice renal artery blood flow times renal venous oxygen content. A multiple linear regression analysis with backward elimination procedure was undertaken to define contributions of the variables to Δ[ScvO_2 _- SvO_2_].

## Results

**Figure 1:**
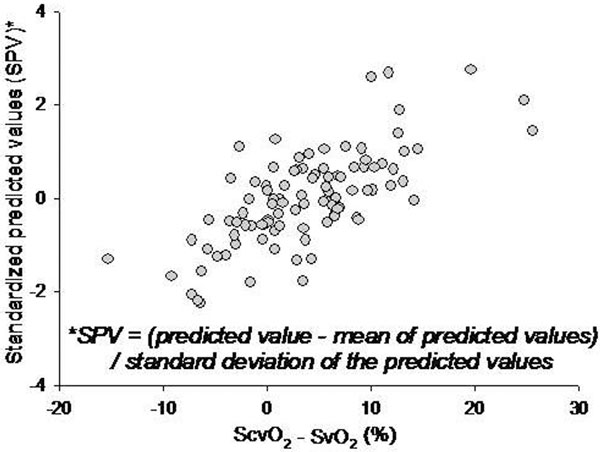
**Scatterplot of standardized predicted values versus ScvO_2 _- SvO_2_**.

Ninety-eight assessments were obtained (one to seven/animal). The backward elimination procedure yielded a best model containing hepatic venous oxygen efflux (*r *= -0.46, *P *< 0.01) and carotid artery blood flow (*r *= 0.56, *P *< 0.01; Figure 1). This final model accounted for 49.8% of variation in Δ[ScvO_2 _- SvO_2_] (*R*^2 ^= 0.498).

## Conclusions

Carotid artery blood flow and hepatic but not renal venous oxygen efflux predict some of the differences between mixed and central venous oxygen saturation. As a consequence, SvO_2 _cannot be predicted by ScvO_2 _alone.

